# A Missing Piece? Men and the Puzzle of Gender Mainstreaming in the European Union

**DOI:** 10.1177/1097184X231192024

**Published:** 2023-08-07

**Authors:** Elaine Weiner

**Affiliations:** 1 Department of Sociology, 5620 McGill University, Montreal, QC, Canada

**Keywords:** gender equality, European Union, politics, feminism, social policy, violence against women, gender-based violence, education

## Abstract

In its early uptake and sweeping application of gender mainstreaming, the European Union (EU) sits in the vanguard. However, bringing a gender perspective to bear on policy has proven a stubborn challenge. Drawing on Bacchi’s “What’s the Problem Represented to Be?” approach and her conceptualization of policies as gendering practices, I critically interrogate how men have been implicated in the problem of gender inequality via policy discourse in the EU. I focus on violence against women/gender-based violence and gender inequalities in education. Analysis of these two issues serves to highlight some of the interpretive limits to the problem of gender inequality in the EU and likely beyond. The discursive elusiveness of men works to keep much of the workings of gender power obscured. Such discounting of “the man question” signals a significant misstep that undercuts gender mainstreaming’s transformative prospects.

## Introduction

The year 2022 marked the twenty-fifth anniversary of the European Union’s (EU) formalization of its commitment to gender mainstreaming.^1^ In its expansive aspirations, gender mainstreaming signaled a major transformation in understanding the problem of and solutions to gender inequality, moving beyond an “add women and stir” approach that focused on select policy areas (e.g., care, employment) that were seen as epitomizing women’s issues, particularly those of cisgender women (Bunch 1990; see also Rees 1998). Gender mainstreaming reconceived the scope of the problem from that of women to one of gender relations—largely between cisgender women and cisgender men—and emphasized the need for men’s involvement in its righting. While the call for its worldwide application, put forward in the *Beijing Declaration and Platform for Action* at the fourth UN World Conference on Women (1995), inspired numerous countries and supranational bodies to endorse gender mainstreaming, the EU sits in the vanguard. Having played an instrumental role at the Beijing conference, particularly “… in formulating the final declaration and action,” the EU took the development of its “approach to equality” as “the basis for action both in the [European] Community and the rest of the world” (European Commission 1996, 2). In adopting gender mainstreaming on a “comprehensive scale,” the EU solidified its global stature as an exemplar in the promotion of gender equality (Blofield and Haas 2013, 711).

Despite the momentum for change that the Beijing Platform for Action seemingly inspired, gender mainstreaming’s transformative promise has yet to be realized in the EU. Indeed, several scholars have characterized the task of mainstreaming gender in EU policy as rather “Sisyphean” (Benshop and Verloo, 2006; Meier and Celis, 2011; Weiner and MacRae, 2014). Though scholars have identified an array of impediments, the fundamental question of men’s integration into EU gender equality efforts has drawn little of their regard (Hearn et al. 2021; Scambor et al. 2014). In this article, I *go back to Beijing* to consider *Whither Men*? in the EU’s gender equality strategy. Empirically, I cast my analytic lens on two types of policy documents—the EU’s multi-year policy action programs and the annual reports on equality between women and men in the EU—that enable an overarching view of the EU’s gender equality framework since the Beijing Platform for Action’s global call for gender mainstreaming. I focus on two policy issues that sit consistently among the five (sometimes six) “priority areas” identified in the EU’s policy action programs on gender equality, put out every few years. The first, variably referred to as “violence against women” (VAW) and “gender-based violence” (GBV), has historically been esteemed a “woman’s issue” and more recently has become politically fraught over the concept of gender employed in the Istanbul Convention. The second, educational inequality, constitutes a realm in which the gender imbalance has tilted, to some extent, in boys’/men’s disfavor.

I take inspiration from Bacchi’s (1999, 2009, 2017) “What’s the Problem Represented to Be?” (WPR) approach and her more recent conceptualization of policies as “gendering practices” to critically interrogate how men have been implicated in the problem of gender inequality since the EU’s adoption of gender mainstreaming. Bacchi’s WPR approach emphasizes the need to consider the interpretive bases that inform policy discourse as well as the implications of—and alternatives to—these interpretations. Discourse, here, represents a core modality through which power relations, gender and otherwise, are “exercised, reflected, maintained and resisted” (Lazar 2005, 11). In conceptualizing policies as “gendering practices,” Bacchi (2017) avers that policies “‘do’” gender and advocates analyzing policy discourse towards “captur[ing] the ways in which inequality is ‘done’” to “interfere” with it (20). As Hearn and McKie (2008) further emphasize, “recognizing the interconnections of gender and policy … means gendering men as an explicit part” of policy formulation (78). Accordingly, I incorporate Messner’s (1997) tripartite framework developed to make sense of “masculinity politics” in “varying discourses and actions” that emphasizes men’s structural/institutional privileges, differences/inequalities among men, and the costs of (hegemonic, narrowly defined) manhood (22). Messner’s conceptual triad works to clarify the discursive markings of “the man question.”

Despite the Beijing Platform for Action’s rousing call for integrating men, substantively and manifestly, to effect gender-conscious (and equalizing policy resorts), my analysis suggests that such integration, in the EU case, remains more stated ideal than policy praxis. I find that women and their victimization take up most of VAW/GBV’s discussion, with perspicuous consideration of men decidedly scarce. Policy talk on educational inequalities, although acknowledging declines in boys’ participation and performance, has similarly yet to significantly alter the interpretive incline toward girls in favor of a more gender-balanced read of the issue. In the gendering of these two policy problems, men are not fully recognized or problematized. These limiting representations of men obscure gender power relations as well as other power asymmetries (e.g., race/ethnicity). The discursive exclusiveness of men works to keeps “alterations in the asymmetrical status of social groups within limits” (Bacchi and Eveline 2012, 118). Such discounting of “the man question,” in feminist pursuit, I suggest, signals a significant misstep that impedes gender mainstreaming’s transformative possibilities because without *seeing* men, gender power relations are not wholly up for challenge.

## Mainstreaming Gender in the EU: Whither Men?

Albeit deemed the Common Market, the EU’s foundational ambition to promote market growth and competitiveness did not reflect a common interest. To the contrary, it by and large advanced the interests of men, particularly cisgender men in positions of political and economic power. While in the 1970s and 1980s the EU began to actively promote the equal opportunity/treatment of women and men in the labor market and positive action measures such as flexible hours, the redress of inequality was narrow in scope—confined primarily to the domains of employment and social policy and designed principally to promote cisgender women’s participation in the labor market. Such efforts to fit women into the “market,” as workers alongside men, functioned considerably to mobilize and orient feminist EU studies. The scholarship that ensued focused predominately on women and the effects of the EU’s various equalizing tactics on women’s paid labor prospects and their care responsibilities (Guerrina 2005; Hoskyns 1996; Rubery, Smith, and Fagan 1999).

By the 1990s, gender mainstreaming—initially borne out of an effort to re-envision development policy in the developing world—had moved onto the global stage, with the Beijing Platform for Action calling for its all-purposing irrespective of policy or place. For the EU, the Beijing conference seemingly inspired a momentum for change towards the assumption of a holistic gender perspective to advance gender equality.^2^ Sweden and Finland, in their EU presidency programs of 2001 and 2006, respectively, elected to highlight “the matter of men’s involvement [in gender equality endeavors].”^3^ In 2006, the European Commission’s Advisory Committee on Equal Opportunities for Women and Men put forward an *Opinion* on “Men and Gender Equality” that contained numerous recommendations “to advance the issue of men in gender equality” (4). In the *Opinion,* the committee advised mobilizing men towards “challenging and changing the structures, institutional policies and practices, and culture” that underlie gender inequality, advancing “new models of masculinity and new thinking about and expectations of men, men’s behaviour and the role of men” and attending to men’s specific needs as men (e.g., health provision) (4). However, the European Institute for Gender Equality, the EU’s support agency for gender equality, declared in a 2010 *Analysis Note* on men and gender equality that a “framework for action on men should be developed as part of an integrated gender equality strategy,” suggesting that gender mainstreaming was not so ready an endeavor in terms of bringing men in (1, 12). A 2013 study undertaken for the European Commission’s Directorate-General for Justice (responsible for the EU’s policies on gender equality) similarly signaled a disconnect between intent and action in its recommendation that a “balanced and coherent “men and equality” policy embedded in gender equality policies” be developed (Scambor, Wojnicka, and Bergmann 2013, 12).

Despite a dynamic start, gender mainstreaming has struggled to gain traction in the EU. Gender mainstreaming’s aspiration to bring gender to bear everywhere spurred feminist EU scholars to query an ever-growing range of policy fields such as climate change (Allwood 2014, 2020), economic governance (Elomäki 2023) migration (Mushaben 2011), rural development (Bock 2015) and science and research (Abels 2011; Mergaert and Lombardo 2014). This scholarship has identified a multitude of challenges to gender mainstreaming such as its conceptual ambiguity, lacking political will, the downgrading of gender mainstreaming (relative to other political and/or economic priorities) and an overreliance on “soft” incentives (Cavaghan 2017; Elgström 2000; Hafner-Burton and Pollack 2009; Lombardo and Meier 2008; Rees 2005). For example, Allwood (2014) points to resistance from powerful institutional actors (e.g., Council of the European Union) for gender-blindness in EU climate change policy, and Bock (2015) characterizes gender mainstreaming in EU rural development policy as a little more than a “tick box” exercise. Feminist EU studies scholars, however, have continued to equate gender equality with “women’s inclusion”—especially cisgender women—to a significant extent and thus have largely steered clear of “the man question” in their calculation of gender mainstreaming’s deficits (Ellerby 2017).

Over the past 25 years, feminist EU studies has focused on women seeking to gain ground in the formal and informal workings of the EU. This has meant, for instance, considering the informal interactions and alliances between feminist scholars, feminist activists, femocrats and EU policymakers (Holli 2008; Woodward 2004). It has further entailed investigating women’s representation and participation in EU decision-making positions and processes and probing the “institutional power play[s]” behind the effort to advance gender equality in the EU (Ahrens 2016; Ahrens and Rolandsen Agustín 2019; Elomäki and Kantola 2020; Kantola 2009).

Some feminist EU studies scholars have directed their analytic gaze towards the EU’s broader anti-discrimination agenda, set in motion in 1997, which situates gender among multiple discriminatory grounds (Krizsán, Skjeie and Squires 2012). Such work generally considers the challenges involved in attending to the “diversity of women’s interests” and the implications of attending to multiple and intersectional inequalities for gender equality objectives (Lombardo and Verloo 2009; Rolandsen Agustín, 2013, 5). Most recently, some have sought to make sense of a growing opposition to women and LGBTQ rights from varied constituencies—in Europe as well as more globally—that names “gender ideology” (sometimes termed “gender theory”) as an “attack on either nature (religious actors), nation (nationalist actors) or normality (conservative actors)” (Kuhar and Zobec 2017, 36; see also Kuhar and Paternotte 2017; Verloo 2018).

The EU has decidedly grown its approach to gender equality over the past half-century, from tactics that initially targeted cisgender women, to the redress of gendered power relations via gender mainstreaming and the introduction of a new politics of equality combatting discrimination on multiple fronts. Nonetheless, feminist EU studies scholars continue to lament the arduousness of pulling up the patriarchal stakes that pin down EU politics. From its inception, feminist EU studies has foreground “the woman question,” despite the recognition that men are a key piece of the gender power puzzle. Gender mainstreaming heralded a major turning point for the EU in its insistence on a clear sighting of women *and men* as gendered beings in relation to each other and in its all-encompassing policy reach. The European Commission’s (1996) initial communication about gender mainstreaming in the EU plainly underscored these two objectives, necessitating “actively and openly taking [a gender perspective] into account” and “mobilizing all general policies and measures specifically for the purpose of achieving equality” (2). In its official adoption of gender mainstreaming, the EU seemingly endorsed a call for change in approach that problematized men, deliberately and overtly, as gendered subjects in the policy process, with gender mainstreaming’s transformative promise resting on the uptake of such reform.

Despite the Beijing Platform for Action’s invocation to engage “the man question,” feminist EU studies, has largely maintained its focus on women and especially cisgender “women’s concerns,” albeit giving way to consider some intersectional inequalities (Connell 2005, 1805). When internal intimations emerged, in subsequent years, that much remained undone or uneven in the EU’s efforts to reform its approach in terms of bringing in men; even so, “the man question” remained of marginal interest in feminist EU studies, with comparatively few scholars moving men and masculinities forward for overt scrutiny in EU policy processes (Klatzer and Schlager 2011; Kronsell 2016a, 2016b; Prügl 2011). How “actively and openly” men are accounted for “in all general policies” since the EU’s adoption of gender mainstreaming remains unclear (European Commission 1996, 2). As Hearn et al. (2018) assert, “often, men and masculinities, have been implicitly named and implicitly gendered in policy interventions” (56). Men and masculinities remain underproblematized and stubborn to change (Bacchi 1999; Hearn 1998b, 2015; Sanders and Mahalingam 2012). Resisting the “gender hierarchy” requires “taking gender seriously and engaging with men more systematically” in ways that attend to the complexity of men and masculinities (Hebert, 2007, 41; see also Dowd 2010). Gendering policy, contend Hearn et al. (2018), necessitates both the “naming of men” and the “critical gendering of men” explicitly (55).

## Data and Method

Since the early 1980s, the European Commission has put forward what are variably termed action or work programs that set out multi-annual (3–5 year) priority policy actions on equality between women and men and serve as a basis for cooperation between the European Commission, other EU institutions (e.g., Council of the European Union) and various other stakeholders. These programs are monitored and evaluated midway (e.g., *Midterm Progress Report on the Roadmap for Equality between Women and Men 2006–2010*). Additionally, in 1996, the European Commission began generating annual reports regarding equality between women and men that assess progress achieved and layout future policy orientations.^4^ These multi-year programs, midterm reviews and annual reports are not pieces of legislation or directives. They are largely agenda-setting mechanisms through which the problem of gender inequality is expressed.^5^ They convey “constructed forms of social knowledge” that produce social problems and “political subjects” (Payne 2014, 957). They are directed at a broad readership that includes the European Parliament and other EU institutions, member state administrations, non-governmental organizations and gender equality experts. It is not until the *Fourth Medium Term Action Programme on Equal Opportunities for Women and Men (1996–2000)* that gender mainstreaming makes it on to the agenda as a “principle” to be elaborated. According to the second annual report, *Equal Opportunities Between Women and Men: Annual Report 1997*, “The real significance of 1997 will become clearer as the Union comes to implement the Amsterdam Treaty after ratification [in 1999] as the logic of mainstreaming is brought to bear in [an] ever widening field of policy areas” (7). I therefore analyze all multi-year policy programs and annual reports realized from 2000 through 2020.^6^

I empirically consider *how* men achieve reference—and their mutability over time—in the discursive character of the problem of gender inequality as articulated in these communications (Bacchi 1999, 2009, 2017; Messner 1997). My analysis relies on three of six prospective lines of inquiry from Bacchi’s WPR approach: first, the “problem” represented; second, that which remains unproblematized in this representation; and third, the effects ensuing from such representation (Bacchi 2012). I used MAXQDA qualitative data analysis software to code all the texts. Bacchi’s core query, “What’s the problem represented to be?” informed my first-order codes. Thus, I coded the identification and discussion of the array of policy problems manifest and their gendering. For example, labor market access achieved repeated mention as a problem for older women and thus, I coded it accordingly Health was intermittently deemed a problem for both women and men and therefore, I coded it in line with its gendered reference. I subsequently carefully re-examined first-order code data, using Messner’s (1997) conceptual triad, to consider: (1) how men’s institutionalized privilege/power relative to women is recognized; (2) how men’s versus women’s institutionalized privilege/power (or lack thereof) is complicated via interaction with other forms of inequality (e.g., class, sexuality); and (3) to what extent, if any, are ensuing losses along the lines of gender made manifest. My second-order coding deductively distinguished these elements.

There is a resulting myriad of possible departure points to consider men’s representation in the problematizing of gender inequality in the EU. My analysis focuses on two particularly compelling sites: VAW/GBV and “Gender Inequalities in Education.” While VAW/GBV both circulate, their core conceptual emphases differ, with GBV, of late, becoming a site of considerable contestation (Kriszsán and Roggeband 2021). The latter, “Gender Inequalities in Education,” poses a challenge to the predominant woman-centric rationale for gender inequalities’ redress due to a partial reversal, in recent decades, of the gender gap in Europe, with boys now falling behind girls in educational attendance and achievement. This about turn has moved boys out of the background, with boys widely touted as in crisis (Farrell and Gray 2018).

## Violence against Women/Gender-Based Violence

Prior to the introduction of gender mainstreaming, the only official document on VAW was a resolution, adopted by the European Parliament in 1986, calling mainly for data collection about its incidence and financial cost.^7^ The EU long lacked any “legal competence in the domain of violence”; however, since 1997, it has relied on various means to combat VAW, the most prominent being the DAPHNE program started in 2000 (Hearn, Pringle, and Balkmar 2016, 554). DAPHNE provides support to a host of non-governmental organizations toward the combatting and prevention of violence against women. In 2017 the EU signed the “The Council of Europe Convention on Preventing and Combatting Violence Against Women” (also referred to as the Istanbul Convention) which sets legally-binding standards on the prevention of violence (on the basis of gender), including the protection of its victims and prosecution of its perpetrators. The EU’s accession to the Convention, has yet to occur however, due to contestation from several member states over the term “gender” and its interpretation of VAW as GBV. As the 2019 *Annual Report* explains, “Some see these concepts as threatening ‘traditional’ family values”; it further points to “various views on the concepts of gender and sex” that are “often misunderstood” (45).

While, in theory, VAW and GBV are conceptually distinct from one another, they both circulate in EU gender equality policy talk, often without clear distinction. This terminological slippage works to narrow GBV definitionally to VAW, with gender, in effect, “flattened to the woman’s question” (Lomazzi and Crespi 2019, 23). A subsection of the 1998 *Annual Report* titled, “Gender-Based Violence,” captures this co-mingling*,* with the immediately ensuing text going on to emphasize that “The [European] Commission holds the view that issues relating to violence against women should be integrated into European Union policies, in the field of external relations and foreign aid, and also in a number of other policy fields…” (106). This conceptual slippage between gender and women carries on in more recent years, with the *Strategic Engagement for Gender Equality 2016–2019* remarking on the “many forms” of GBV; however, all the forms mentioned (physical violence, sexual violence, online harassment, female genital mutilation), with the exception of human trafficking, are discussed with reference to only women’s risk or experiences (8). In the most recent policy action plan, *A Union of Equality: Gender Equality Strategy 2020–2025,* the blurring of gender and women continues: “Gender-based violence—or violence that is directed against a woman because she is a woman or that affects women disproportionately—remains one of our societies’ biggest challenges and is deeply rooted in gender inequality” (3).

VAW/GBV holds a rather waving foothold in the EU’s policy action programs and annual reports on gender equality between 2000 and 2010. In *Towards a Community Framework Strategy on Gender Equality 2001–2005,* the discussion of VAW/GBV falls under the priority area of “gender equality in civil life.” In its successor, *A Roadmap for Gender Equality 2006–2010,* the “eradication of all forms of gender-based violence” represents a priority area (1 of 6). There is, however, hardly any discussion of VAW/GBV between 2000 and 2009 in the annual reports, with those pertaining to 2001–2004 sparingly mentioning domestic violence and the 2005–2009 reports containing the term “violence” a cumulative total of four times. From 2010 onward, VAW/GBV consistently figures among the priorities for EU action to combat gender inequality and to insure women’s human rights, with reference to life, dignity, and security.

The explicit marking of women and girls, as the principal victims of violence—abused, bullied, enslaved, harassed, mutilated, raped, stalked, trafficked, etc. —remains a constant framing in EU gender equality policy action programs and annual reports, with scant specification as to residual victims. The mention of men and boys among a breakdown of human trafficking victims in the *Strategic Engagement for Equality 2016–2019* constitutes an anomalous instance: “women make up the majority of human trafficking victims (68% women, 17% men, 12% girls and 3% boys)” (3). The coupling of men and/or boys with victim status remains decidedly rare and when such victimization is extant, it is discursively inconspicuous. The victimization of women (as well as girls), however, has remained rather tautologically accounted for—“experience[d] because they are women” (*Strategy for Equality Between Women and Men 2010–2015*, 80). This rendering of cause-effect offsets culpability from women, making a postulation of women as instigators virtually inconceivable. Concomitantly, it offers little to no explanatory power for men’s violent behavior. More generally, however, men are seldom named as perpetrators of VAW/GBV—whether as abusers, rapists, traffickers and so forth. The wrong (i.e., violence) must be stopped (prevented) and that from which women must be safeguarded (protected); the wrongdoer goes unspecified. This partial obfuscation of the gendering of the policy problem of VAW/GBV deters its dismantling.

Men’s role in or responsibility for such violence goes unspoken. An association in the 2010 *Annual Report* represents the exception: “The majority of these violent acts [i.e., physical and sexual] are carried out by men in their immediate social environment, most often by partners and ex-partners” (12). The assumed interaction between intimate partners or former partners, in this EU communication, points toward a limited interpretation of violence as intimate, occurring for the most part in heterosexual couplings, with an essentialist understanding of women and men. The same *Annual Report* does go on to note the “serious social and financial consequences, with high costs for the health sector, social services, the police and judiciary and for the labor market” of such violence (13). With men’s privilege and power institutionalized across these realms, men’s interests are inherently at stake. Yet, by and large, men’s institutionalized power provokes no direct questioning or challenge. Peril to, rather the problem of, patriarchal power achieves emphasis here.

The narrow scope of VAW/GBV has proven quite steadfast despite growing pressure to define the issue more comprehensively, considering alternative modes of gender violence, particularly that transpiring between men (Hearn 1998a). Man-to-man violence involves the production and maintenance of an internal gender hierarchy: “… among the male victims of interpersonal violence (performed by other men), the majority come from so-called marginalized, nonhegemonic and nondominant social groups” (Scambor et al. 2014, 568). Man-to-man violence such as military conflict further broadens the parameters of gender-based violence in terms of *where* (public space) and among *who* (unknown persons) such violence arises. While the entrée of “cyber violence” (e.g., cyberstalking, sexual harassment)—emerging initially in the 2013 *Annual Report* and taken up in subsequent annual reports—does widen out the potential *wheres* and *whos* of gender-based violence, no consideration of its broader gendered possibilities (e.g., between cisgender and transgender persons) comes about. There is one acknowledgement in the 2017 *Annual Report* of gender-based violence as including “… violence in close relationships as well as street violence…” but there is no definition of street violence, its gendering, or any future mention. Additionally, women who are public figures glean mention in the 2018 *Annual Report* discussion of “cyber violence”—as a “phenomenon of recent concern”—with women politicians deemed to “experience a double burden created by their public status as well as their gender, while human rights defenders are more often attacked when they are women” (38). Whether as offenders or victims, overt scrutiny of men’s power—or the relative lack thereof—is obscured in the discussion of VAW/GBV. Such slanted and superficial consideration of men presents little threat to the inequitable status quo.

Since 2010, Roma women, women with disabilities and migrant/refugee women have elicited some naming for their heightened susceptibility to VAW/GBV. Much of this nuancing, however, seems more reactive—spurred, for instance, by the migration surge in Europe that peaked in 2015—versus a systematic appreciation of the ways in which social factors (e.g., race/ethnicity) impact their vulnerability. The ethnic labeling of Roma, migrant and refugee women also risks functioning as a more “exclusionary” than “inclusionary” way of “dealing with difference,” with “culture” blamed for the violence “perpetrated by and against members of certain societies and social groups” (Montoya and Rolandsen Agustín 2013, 538). Men’s part, as victims or perpetrators, remains unsaid in the EU’s policy action programs and annual reports on gender equality. For instance, the 2012 *Annual Report* affirms, “Roma women often face multiple forms of discrimination including within their own communities …. Many of them become victims of human trafficking, sexual abuse and enforced prostitution” (24). Though the *where* of Roma women’s victimization implicates “their own communities,” *who* victimizes, whether Roma men or other men, is not identified with any explicitness. Human trafficking, sexual abuse and prostitution are, in a sense, enlivened as the victimizing *who*—able to enact harm on Roma women.

VAW/GBV’s women-centric lens renders men barely visible in the policy problem expressed via EU policy action programs and annual reports on gender equality. While men exact VAW/GBV and can also be a victim of it, men draw minimal discussion. The longstanding “prevention, protection and punishment” refrain to this problem foregrounds women’s need for protection but leaves the person for punishment unspecified. Men’s institutionalized power (or its qualification) is talked around—an evasion that does not, straightforwardly, or steadily, advance its unsettling. According to the 2014 *Annual Report*, the EU’s Agency for Fundamental Rights carried out the first survey on VAW, interviewing more than 24,000 women; no men were surveyed. Yet, as the report avers, this data will serve as a “basis for gauging the right policy responses” (22). Here, men’s gender interests are either presumed, derived from those of women or wholly passed over.

## Gender Inequalities in Education

Since the 1970s, the disadvantages girls faced relative to boys in terms of participation, performance and field of study preferences defined the meaning of the gender gap in education not only in the EU but well beyond. In the EU, rectifying these inequities involved concerted efforts to elevate girls, educationally, to the level of boys. Policy measures to effect change reflect considerable success, particularly in terms of attendance and achievement. The educational attainment of girls and women as surpassing that of boys and men, especially at the secondary and tertiary levels but also in terms of lifelong learning (i.e., adult education and training), draws repeated favorable remark from 1998 onward in the EU’s annual reports on gender equality. However, this is repeatedly qualified in the reports as not fully carrying over into alleviating strong sex stereotyping in field of study preferences that are transmitted, in turn, into the labor market. The consequences of this carryover are largely referenced as consequential for women and a depersonalized economy via the “undervaluation of women’s work … suboptimal matching of skills and jobs, and … bottlenecks on the labor market” (*Annual Report* 2014, 13). Though, the privileged overvaluation of men’s work and its labor market significance goes undeclared.

Combatting such stereotypes in education, in the name of equality and the economy, has constituted a priority action since 2001. In the EU’s gender equality policy framework, the eradication of such gender biases in educational curricula along with training and guidance for girls and boys has long been cast as necessary to change girls’ and women’s educational and occupational choices such that they are encouraged to explore non-traditional fields, particularly STEM studies. Put differently, girls’ and women’s unlikely choices are the featured predicament. Though *A Roadmap for Gender Equality 2006–2010* cited the imperative for education and training policy that encourages “young women and men to explore non-traditional educational paths,” boys’ and men’s averted decisions away from feminized educational or employment paths are virtually never examined in the EU’s gender equality policy action programs or annual reports (8). A fleeting link to boys and the constraints of educational gender biases in the 2012 *Annual Report* highlights the need to implement policy reforms “to tackle gender inequalities at an early stage and so ensure that all boys and girls can realize their potential and choose the field they are good at, without being limited by prejudice” (16). The interference that “prejudice” poses, however, remains represented principally as weighing down women, with the onus put on women to readjust towards achieving a “better gender balance across studies and occupations” (*Annual Report* 2009, 8). Even as of the 2019 *Annual Report*, a discussion of the social construction of career choices considers solely the potential factors shaping women’s professional pursuits. Only very recently has men’s limited labor market mobility moved slightly into the problem purview, appearing in the 2018 *Annual Report,* with “underpayment” deemed “a form of inequality” that lessens men’s motivation to enter and remain in women-dominated occupations (23).

The rather tenacious “preference” problem and more precisely, its disadvantaging of girls and women, functions as the lodestone in EU policy talk on gender inequalities in education. The “phenomenon” of early school leaving, which “affects more boys more than girls” does, however, also draw regular mention in the EU’s multi-year gender equality policy action programs (*A Roadmap for Gender Equality 2006-2010*, 8).^8^ In more recent years, these communications give a nod to the bearing of other forms of inequality, albeit variably vague to quite exacting in their reference. The *Strategic Engagement for Gender Equality 2016–2019* notes, “Boys, especially from disadvantaged backgrounds drop out of school more than girls,” though the nature of disadvantage is never specified (7). Roma girls receive a somewhat capricious co-optation, mentioned, at one point, in the midterm review of the *Strategy for Equality Between Women and Men 2010–2015*. The 2003 *Annual Report* and the *A Roadmap for Gender Equality 2006–2010*, identify access to schooling itself as a difficulty “Muslim women” and “migrant women” face, respectively. There is, however, no systematic engagement of intersectional considerations over time in EU policy talk on gender inequalities in education; to the contrary, there is seldom any mention at all. In the *Annual Report*s, early school leaving mainly achieves emphasis via a single bar chart on “early school leavers” (by gender and country) for the reporting year that is in the statistical annex of annual reports. Aside from the chart, the annual reports rarely mention boys’ and men’s school leaving. Indeed, only in 2013 and 2014 does boys’ greater likelihood of dropping out of school relative to girls achieve mention in the body of the reports. Interestingly, in the 2012, 2013 and 2014 annual reports, the rates of school leaving are compared with 2002 and 2008 reported rates, revealing considerable progress, though concerning gaps remain. Yet, after 2014, the annual reports no longer include the chart nor is there any discussion of this gender gap.

Boys’ lag in participation and performance in school has worked, to some degree, to better implicate boys and men in the problem of gender inequalities in education. The partial sighting of boys is encouraged by the advance of girls that is growing a gap whose inequity runs counter to most social and economic inequalities between girls and women and boys and men. Such an obvious incongruity may account for its notice. Nonetheless, a lopsidedness prevails in how boys and men factor in. Though both girls’ and women’s and boys’ and men’s skewed educational choices are recognized in the problem, the educational preferences of girls and women and more specifically, their turn away from STEM fields are accentuated. Across the EU’s policy action programs and annual reports on gender equality, the promised returns of removing such gendered constraints are further made far more explicit for girls and women than boys and men. The derivatives, positive or negative, for boys and men, with respect to altering the educational status quo, are more oblique to nonexistent. Effectively here, boys’ and men’s institutionalized privilege is recognized but seldom problematized, with their education and resultant occupational paths seldom directly troubled. Over time, the issue of boys’ dropping out of school seems to have somewhat dropped out in terms of its problem presence. Moreover, the interplay of other forms of inequality with that of gender here proves scarce and scattered.

## Discussion and Conclusion

[P]rogress is moving forward at a snail’s pace and in some domains is going backwards … the EU is still a long way from a gender equality society.

—Annual Report, 2018

The latest work program, *A Union of Equality: Gender Equality Strategy 2020–2025* highlights the recent and novel appointment in 2019 of a Commissioner for Equality with a “stand alone portfolio” (15). It further announced the creation of a Task Force for Equality that will “ensure the implementation of equality mainstreaming, including gender equality” (15). These two actions appear to signal the EU’s desire to fortify and “enhance” gender mainstreaming (2). The strategy foregrounds a “dual approach,” involving the “systematic” inclusion of a “gender perspective in all stages of policy design in all EU policy areas”—and its implementation “using intersectionality…as a cross-cutting principle” (2). Additionally, the expression “in all their diversity” is introduced “to express that, where women or men are mentioned, these are heterogeneous categories” (2). Such language, designed to “affirm the commitment to leave no one behind and achieve a gender equal Europe for everyone,” seems to signal an earnest move towards a more inclusive and consistent regard for how the “combination of gender with other personal characteristics or identities” has a bearing in the problem of gender inequality (2).

Despite the enthusiastic reception of gender mainstreaming that the Beijing conference seemingly inspired in the EU and beyond, effecting gender-conscious policy remains a thorny enterprise. A formidable and ever-growing literature has emerged, focused on varying facets of the EU’s trajectory towards gender equality. There is little contention among feminist EU studies scholars as to the intractability of the problem of gender inequality in the EU, with an array of ideational and institutional mechanisms identified as impediments. However, “the man question” has remained outstanding, despite Beijing’s rallying cry for men’s integration to ultimately achieve gender equality.

My analysis returns to this collective call and its paradigmatic shift in terms of conceptualizing both the problem of and solutions to gender inequality. I treat EU policies as “gendering practices” to tease out *what* is problematized in terms of gender inequality and *who* is subjectified, with a particular emphasis on how men are manifest (Bacchi 2017). I bring Bacchi’s (1999, 2009) WPR approach, in part, and Messner’s (1997) tripartite framework (i.e., privilege, differences, costs) on the politics of masculinity to bear to explore the representation of men in the discursive contours of the problem of gender inequality since the EU’s adoption of gender mainstreaming. Analysis of two issues, VAW/GBV and gender inequalities in education, highlights some of interpretive limits to the *problem* of gender inequality in the EU (and likely beyond), revealing that disruption to “the dynamics of gender power (and the structures that support these dynamics)” constitutes a lasting challenge (Hearn and McKie 2008, 79).

Somewhat paradoxically, “much of what is called politics … and public discourse more generally has been centrally about men” and yet, men glean limited reference in these two gender inequality issues (Hearn 1998a, 1). Such a dearth of discourse is not without consequence. Hearn contends that “not explicitly talking of men, not naming men is a structured way of not beginning to talk of and question men’s power in relation to women, children, young people, and indeed other men” (1998b, 786; see also Hearn, Pringle, and Balkmar 2018). It is not forcefully declared; it works more subversively, concealed as that which is “unremarkable” (Sanders and Mahalingam 2012, 115). Its form is “seemingly innocuous” (Lazar 2005, 9). This discursive damper works to both “reduce conflict around structural inequality” and preserve existing power hierarchies (Sanders and Mahalingam 2012, 112; see also Bourdieu 1977; Lukes 2005).

Moreover, despite the “patriarchal dividend” in the form of status, authority, and material resources that men accrue collectively, the returns are not distributed equally among men (Connell 1995). Other mechanisms of marginalization can undercut the coherence of men’s privilege. Though, as Coston and Kimmel affirm, “when discussing privilege, we often consider it a zero-sum quantity, one either has it or one does not” (2012, 97). Men continue to be dealt with as if “unproblematic” and “undifferentiated” (Hooper 2001, 42). Some have further pointed to the “contradictory experience” or “by-products” of privilege, suggesting that it has its costs, emotional and psychological (Kaufman 1999, 59; Pease 2010, 103). “Internalized domination” is unexternalized—the experience muted (Case, Iuzzini, and Hopkins 2012, 3). As Connell (2005) articulates:

“One could draw up a balance sheet of the costs and benefits to men from the current gender order. But this balance sheet would not be like a corporate accounting exercise where there is a bottom line, subtracting costs from income. The disadvantages … are, broadly speaking, the conditions of the advantages” (1809).

With privilege largely tacit or a taboo topic, little occasion exists for the “decentering” of men or a proper reckoning with its complications (Hearn 1998b). Such reductionism muddies which men and women wield power and as a result, impedes its dismantling. Power “… is simply reproduced” (Kronsell 2016a, 104).

The faulty logics guiding gender equality policy efforts remain remarkably resilient in the EU and likely beyond, despite their international denunciation at the 1995 Beijing conference. If the policy problem so fundamentally obscures men, then both the “diagnosis” and the ensuing policy treatment miss their mark (Bacchi 1999, 199). Challenging gender inequalities, here, still connotes “addressing women’s concerns,” and treating cisgender women as “the subjects” versus critically confronting the workings of gender power (Connell 2005, 1805; Ellerby 2017).

The mandated move from “elevating women” to interrogating and overhauling gender relations remains more espoused intent than established practice (Runyan and Peterson 2014, 13). The need, however, to better integrate men into the EU’s gender equality strategy has become increasingly pressing. Connell suggests that the “structure” of gender equality policy in which men are left in the “background” can yield “opportunity for antifeminist politics” (2005, 1805). Backlash against women’s rights and gender equality has not remained a hypothetical in the EU.^9^ The transformative potential of gender mainstreaming remains largely left to the imagination. However, as Bacchi maintains, policy problems are “created by the policy community”; as such, their interpretations are mutable (1999, 199).

## Notes

1. See Article 2 of the Treaty of Amsterdam (1997).

2. As Hubert and Stratigaki (2016) note: “no definition [of gender mainstreaming] has ever been formally accepted at the EU level …. The EP [European Parliament] prefers the definition advanced by the Council of Europe …” (26). The Council of Europe defines gender mainstreaming as “the (re)organization, improvement, development and evaluation of all policy processes, so that a gender equality perspective is incorporated in all policies, at all levels and at all stages by the actors normally involved in policymaking” (1998, 5).

3. Excerpted from Sweden’s EU presidency programme, https://www.europarl.europa.eu/meetdocs/committees/empl/20010123/en.pdf.

4. For some examples of action plans and annual reports, see https://ec.europa.eu/info/policies/justice-and-fundamental-rights/gender-equality_en.

5. Though some taking stock of legislation and directives can exist in these communications, their assessment functions primarily as part of the bounding of the problem. However, as Ahrens (2018) contends, “When investigating gender equality policy at a supranational EU level, it is crucial to analyze Commission communications as the standard policy instrument of the EU. In terms of their direct supranational impact on member states, EU gender equality policy programmes rank particularly high on the list of measures, although they are rarely acknowledged as such” (5).

6. In 2003, 2015 and 2020, no annual reports were published.

7. European Parliament (1986) *Resolution on Violence Against Women* OJ C 176/73 (doc A-2-44/86).

8. Early school leaving, in the EU’s usage, refers to individuals between the age of 18 to 24 who have attained at most a lower secondary education (equivalent to eighth grade by North American standards) and who are not pursing further education or training in the four weeks prior to being surveyed. Early school leavers also include those who “dropout” before completing upper secondary education programs (see https://ec.europa.eu/eurostat/statistics-explained/index.php?title=Glossary:Early_leaver_from_education_and_training).

9. See the European Parliament *Resolution on Experiencing a Backlash in Women’s Rights and Gender Equality in the EU,* 13 February 2019, P8_TA-PROV(2019)0111. https://www.europarl.europa.eu/doceo/document/TA-8-2019-0111_EN.html
